# A radiobiological model of metastatic burden reduction for molecular radiotherapy: application to patients with bone metastases

**DOI:** 10.1088/1361-6560/aa5e6f

**Published:** 2017-03-14

**Authors:** Ana M Denis-Bacelar, Sarah J Chittenden, Iain Murray, Antigoni Divoli, V Ralph McCready, David P Dearnaley, Joe M O’Sullivan, Bernadette Johnson, Glenn D Flux

**Affiliations:** 1 Joint Department of Physics, The Institute of Cancer Research and The Royal Marsden Hospital NHS Foundation Trust, London, United Kingdom; 2 Department of Nuclear Medicine, Brighton and Sussex University Hospitals NHS Trust, Brighton, United Kingdom; 3 Division of Radiotherapy and Imaging, The Institute of Cancer Research and The Royal Marsden Hospital NHS Foundation Trust, London, United Kingdom; 4 Centre for Cancer Research and Cell Biology, Queen’s University Belfast, Belfast, United Kingdom; ana.denisbacelar@icr.ac.uk

**Keywords:** radiobiology, dosimetry, radiopharmaceuticals, bone metastases, prostate cancer, molecular radiotherapy

## Abstract

Skeletal tumour burden is a biomarker of prognosis and survival in cancer patients. This study proposes a novel method based on the linear quadratic model to predict the reduction in metastatic tumour burden as a function of the absorbed doses delivered from molecular radiotherapy treatments.

The range of absorbed doses necessary to eradicate all the bone lesions and to reduce the metastatic burden was investigated in a cohort of 22 patients with bone metastases from castration-resistant prostate cancer. A metastatic burden reduction curve was generated for each patient, which predicts the reduction in metastatic burden as a function of the patient mean absorbed dose, defined as the mean of all the lesion absorbed doses in any given patient. In the patient cohort studied, the median of the patient mean absorbed dose predicted to reduce the metastatic burden by 50% was 89 Gy (interquartile range: 83–105 Gy), whilst a median of 183 Gy (interquartile range: 107–247 Gy) was found necessary to eradicate all metastases in a given patient. The absorbed dose required to eradicate all the lesions was strongly correlated with the variability of the absorbed doses delivered to multiple lesions in a given patient (*r*  =  0.98, *P*  <  0.0001). The metastatic burden reduction curves showed a potential large reduction in metastatic burden for a small increase in absorbed dose in 91% of patients.

The results indicate the range of absorbed doses required to potentially obtain a significant survival benefit. The metastatic burden reduction method provides a simple tool that could be used in routine clinical practice for patient selection and to indicate the required administered activity to achieve a predicted patient mean absorbed dose and reduction in metastatic tumour burden.

## Introduction

Bone metastases are a common indicator of distant relapse from various cancers, in particular those arising in the prostate, lung and breast (Mundy 2002). They can lead to complications such as severe pain, pathological fractures and spinal cord compression; which significantly decrease patient quality of life and shorten survival (Coleman 2001). Current treatments for patients with bone metastases are primarily palliative, including the use of analgesics, external beam radiotherapy, bisphosphonates, chemotherapy or molecular radiotherapy with bone-seeking radiopharmaceuticals (Coleman 2001).

Radiation therapy is an effective treatment modality for bone pain palliation and evidence has shown equal pain relief for single and multi-fraction treatments, providing pain relief in up to 80% of patients (Chow *et al* 2012). The biological effects of radiation in a curative cancer treatment are not as relevant to a palliative setting, as low absorbed doses are used for bone pain palliation in contrast to the higher levels of radiation required for tumour eradication. However, recent treatment advances in stereotactic body radiation therapy (SBRT) have enabled the safe delivery of ablative radiation doses with minimal normal tissue toxicity to patients with low number of metastases and oligometastatic disease (Tree *et al* 2013, Saluja *et al* 2016). Molecular radiotherapy with bone-seeking radiopharmaceuticals can also potentially deliver high absorbed doses to the bone lesions cells whilst minimising bone marrow toxicity (Goyal and Antonarakis 2012). A survival benefit has been observed for ^89^Sr-dichloride following 6 weekly cycles of doxorubicin as compared to chemotherapy alone in patients with metastatic castration-resistant prostate cancer (mCRPC) (Tu *et al* 2001). More recently, ^223^Ra-dichloride was shown to improve survival against placebo (Parker *et al* 2013), introducing a shift in patient management from pain palliation to prolonged survival (Liepe and Shinto 2016). The prospect of further significant improvements in survival is underlined by the limited haematological toxicity resulting from current treatments (Bruland *et al* 2006, Parker *et al* 2013) and the practicality of high activity administrations in combination with stem cell transplantation (O’Sullivan *et al* 2002, O’Sullivan 2006). This indicates the potential for more aggressive administrations if a personalised treatment planning approach is used. Such treatments require quantification of image data to facilitate patient-specific dosimetry and the development of models to explain the clinical significance of the calculated absorbed doses.

Available radiobiological models of metastatic control provide the probability of eradicating all the metastases in a given patient (Bernhardt *et al* 2003, 2004). Numerous studies have shown that the skeletal tumour burden (Soloway *et al* 1988, Dennis *et al* 2012, Denis-Bacelar *et al* 2017) is closely related to overall survival. Therefore, a more clinically realistic measure of metastatic control would be to consider the reduction in metastatic burden and prevention of spread of the disease rather than complete cure. The aim of this study was to propose a new method to predict the decrease of metastatic burden for any given patient-specific distribution of lesion absorbed doses following radiotherapy. The application of this methodology in clinical practice was assessed using available clinical data from patients with mCRPC, which reveals a new framework within which the potential for prolonged survival and metastatic tumour burden reduction could be explored.

## Material and methods

Ionising radiation induces DNA damage mainly via single and double-strand breaks, which can lead to cell death. The linear quadratic (LQ) model is commonly used to describe cell kill from tissue irradiation (Dale 1985), where the cell survival fraction is given as:

1 \begin{eqnarray*}\text{SF}(D)={{\text{e}}^{-\left(\alpha D+\beta G{{D}^{2}}\right)}},\end{eqnarray*}

where *α* (Gy^−1^) and *β* (Gy^−2^) are tissue dependent constants used to describe the probability of cell death by double-strand breaks and by two consecutive single-strand breaks to occur respectively. Repopulation effects were not considered.

Considerably lower absorbed dose rates are delivered in molecular radiotherapy as compared to external beam radiotherapy; therefore the contribution from the quadratic component is smaller, as many of the single-strand breaks are repaired during irradiation. To account for the repair of sub-lethal damage from protracted irradiation during a time *T*, the quadratic component of the LQ model is modified by the Lea–Catcheside factor (*G*) (Millar 1991, Gerweck *et al* 2006):

2 \begin{eqnarray*}G(T)=\frac{2}{{{D}^{2}}}{\int}_{0}^{T}\text{d}t\frac{\text{d}D(t)}{\text{d}t}{\int}_{0}^{t}\text{d}{{t}^{\prime}}\frac{\text{d}D\left({{t}^{\prime}}\right)}{\text{d}{{t}^{\prime}}}{{\text{e}}^{-\mu \left(t-{{t}^{\prime}}\right)}}.\end{eqnarray*}

For molecular radiotherapy, with a mono-exponentially decreasing absorbed dose rate which is allowed to decay (*T*  →  ∞):

3 \begin{eqnarray*}G\left(\infty \right)=\frac{\lambda}{\lambda +\mu},\end{eqnarray*}

where *λ* is the effective decay constant which accounts for the physical and biological half-lives of a given radiopharmaceutical, and *μ* is the repair rate constant. Typical sub-lethal damage repair half-times range between 16 min and 3 h (Dale and Carabe-Fernandez 2005).

The biological effective dose (BED) accounts for the effects of the absorbed dose rate and the radiosensitivity and repair capacity of the considered tissue (Fowler 1989):

4 \begin{eqnarray*}\text{BED}=-\frac{\ln \left(\text{SF}\right)}{\alpha}=D\left(1+\lambda \frac{D}{\left(\lambda +\mu \right){}^{\alpha}/{}_{\beta}}\right),\end{eqnarray*}

where the *α*/*β* ratio quantifies the radiosensitivity of a given tissue to changes in fractionation and *Dλ* is the initial absorbed dose rate of the radiopharmaceutical. Typical ratios for tumours and early-reacting tissues including the bone marrow are 10 Gy, whilst late-responding tissues show ratios of 2–5 Gy (Dale 2004). Prostate cancer is an exception, where most studies indicate low ratios of about 1–5 Gy (Brenner and Hall 1999, Fowler *et al* 2001, Wang *et al* 2003, Oliveira *et al* 2012, Dearnaley *et al* 2016). An average effective half-life of 62 h was used (Graham *et al* 1999).

From the LQ model and considering Poisson statistics, the tumour control probability (TCP) expresses the probability of killing all clonogenic cells (*N*_0_) in a given bone lesion:

5 \begin{eqnarray*}\text{TCP}=\exp \left(-{{N}_{0}}\times \text{SF}\right)=\exp \left(-{{\rho}_{\text{c}}}V\times \exp \left(-\alpha \text{BED}\right)\right),\end{eqnarray*}

where *ρ*_c_ is the clonogenic cell density and *V* is the tumour volume.

The absorbed doses required to obtain a TCP of 0.95 depend on the radiobiological parameters used, however these are not known for radionuclides and bone lesions from prostate cancer. The results of this study are based on parameters corresponding to prostate cancer cells, with a clonogenic cell density of 3 · 10^6^ cm^−3^, *α*/*β* of 3.1 Gy, *α* of 0.15 Gy^−1^ and repair constant *μ* of 0.46 h^−1^ (Wang *et al* 2003).

### The metastatic control probability model

The metastatic control probability (MCP) model (Bernhardt *et al* 2003, 2004), has been previously used in molecular radiotherapy (Elgqvist *et al* 2010, Walrand *et al* 2012). In the context of skeletal metastases, it provides the probability of eradicating all the bone lesions in a given patient and it is defined as the product of the *N* individual lesion TCPs:

6 \begin{eqnarray*}\text{MCP}=\underset{i=1}{\overset{N}{{ \Pi }}}\,_{\,}^{{}}\text{TCP}.\end{eqnarray*}

### The metastatic burden reduction model

A new framework for treatment planning is proposed, where the decrease in metastatic tumour burden is predicted as a function of the absorbed doses delivered to multiple bone lesions. If the patient mean absorbed dose (PMAD) is defined as the mean of *N* lesion absorbed doses in any given patient ($\text{PMAD}={\sum}_{i=0}^{N}{{D}_{i}}/N$), the metastatic burden reduction (MBR) following treatment is calculated as:

7 \begin{eqnarray*}\text{MBR}\left(\text{PMAD}\right)=1-\frac{{\sum}_{i=0}^{N}\,{{v}_{i}}\times {{k}_{i}}\left({{D}_{i}}\right)}{V},\end{eqnarray*}

where $V={\sum}_{i=0}^{N}{{v}_{i}}$ is the metastatic burden at baseline calculated as the sum of the volumes for *N* lesions of volume *v*_*i*_, and ${{k}_{i}}\left({{D}_{i}}\right)=\left\{\begin{array}{*{35}{l}} 1, &amp; TCP\left({{D}_{i}}\right)&lt;0.95 \\ 0, &amp; TCP\left({{D}_{i}}\right)\geqslant 0.95 \end{array}\right. $ is a binary parameter that describes the eradicated (*k*  =  0) or non-eradicated (*k*  =  1) status of the *i*th lesion for a given lesion absorbed dose *D*_*i*_. The MBR ranges from 0 (no metastases have been eradicated) to 1 (all metastases have been eradicated).

### Application to clinical data

Clinical data were obtained from 22 patients with CRPC metastatic to bone treated with a fixed administered activity of 5 GBq of ^186^Re-HEDP and with peripheral blood stem cell transplant support during a phase II clinical trial (O’Sullivan 2006). All patients provided written informed consent to take part in the trials, which were approved by the Royal Marsden NHS Trust and The Institute of Cancer Research Ethics committee. Bone lesion dosimetry and skeletal metastatic tumour burden calculations were performed from quantitative sequential SPECT imaging as part of a previous study (Denis-Bacelar *et al* 2017). The delivered patient mean absorbed dose (PMAD_delivered_) was defined as the mean of the absorbed doses delivered to all lesions in any given patient. The relative distribution of lesion absorbed doses and the lesion volumes were used as the basis for the radiobiology calculations.

Calculations of the TCP were performed under the assumption of uniform absorbed dose distributions within the bone lesion and uniform radiosensitivity and clonogenic density across lesions and patients. The patient mean absorbed dose necessary to eradicate all the bone lesions within a patient (PMAD_MCP=0.95_) was obtained by iteratively scaling the relative distribution of absorbed doses delivered to the lesions in a given patient by a target dose scaling factor until MCP  =  0.95.

To assess the impact of the degree of absorbed dose variability between lesions upon the PMAD_MCP=0.95_, different measures of variability were considered; including the range, variance, standard deviation, coefficient of variation and the range scaled by the minimum value.

The patient mean absorbed dose at which the metastatic tumour reduction is 100% approaches PMAD_MCP=0.95_. Metastatic tumour reduction curves were obtained for a range of patient mean absorbed doses derived from scaling the relative distribution of absorbed doses delivered to the lesions for all patients. The patient mean absorbed dose required to decrease the metastatic tumour burden by 50% (PMAD_MBR=0.5_) was calculated for each patient.

To assess the impact of the cut-off probability on the metastatic control and metastatic burden reduction, the calculations were also performed for values of 0.9 and 0.99. The effect of an *α* parameter of 0.25 Gy^−1^ to represent metastatic lesions less radio-resistant than prostate cancer cells was also characterised.

#### Comparison between predicted and delivered absorbed doses

The predicted PMAD_MCP=0.95_ and PMAD_MBR=0.5_ values for the 22 patients were compared with the PMAD_delivered_ from 5 GBq of ^186^Re-HEDP. Follow-up imaging was not available to determine the actual metastatic burden reduction following treatment.

### Statistical considerations

Median and interquartile range (IQR) were used to describe continuous variables. Pearson’s correlation coefficients were used to quantify the degree to which two variables are related and regression analysis was used to identify relationships between variables. Two-sided *P*-values below 0.05 were considered significant.

## Results

### The metastatic control probability model

**Figure 1.**
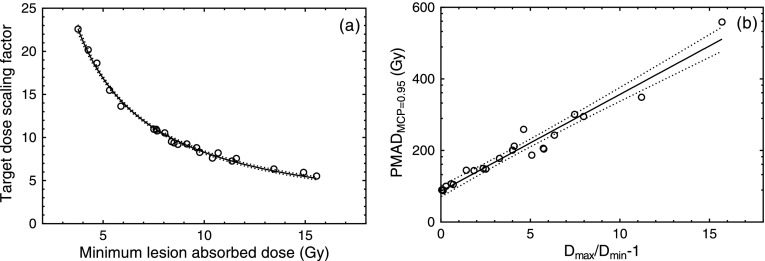
Relationships between the target dose scaling factor and the minimum lesion absorbed dose in a given patient (a), and between the patient mean absorbed dose required to eradicate all the lesions (PMAD_MCP=0.95_) and the variability of the distribution of absorbed doses delivered to the lesions in a given patient represented by the relative difference between minimum and maximum lesion absorbed doses (b). The 95% confidence bands are shown.

A median PMAD_MCP=0.95_ of 183 Gy (IQR: 107–247 Gy) was obtained across the 22 patients. For cut-offs of 0.9 and 0.99 the PMAD median values of 178 Gy (IQR: 104–241 Gy) and 193 Gy (IQR: 113–260 Gy) were obtained respectively. An *α* parameter of 0.25 Gy^−1^ reduced the PMAD_MCP=0.95_ by 32% on average, with a median of 125 Gy (IQR: 73–169 Gy). Figure 1(a) shows a strong negative correlation and power relationship between the target dose scaling factor and the minimum lesion absorbed dose (*r*  =  −0.89, *P*  <  0.0001). This indicates the strong influence of the minimum absorbed dose in the probability of metastatic control.

From all the measures of absorbed dose variability in a given patient, the best correlation with the PMAD_MCP=0.95_ was obtained with the range scaled by the minimum absorbed dose (*r*  =  0.98, *P*  <  0.0001), shown in figure 1(b). When the ratio between the maximum and minimum lesion absorbed dose increases by an order of magnitude, the patient mean absorbed dose required to eradicate all the lesions increases more than 300% (from 49 Gy to 200 Gy for a ratio of 1–10 respectively), highlighting the negative effects of absorbed dose variability between lesions.

### The metastatic burden reduction model

**Figure 2.**
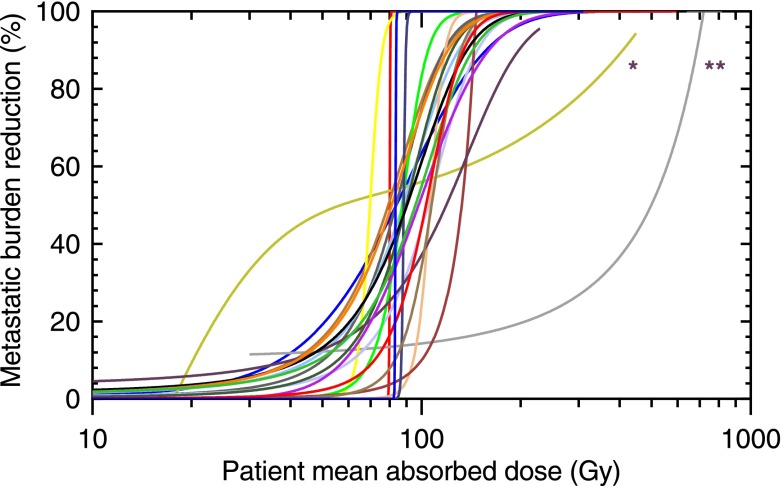
Predicted metastatic burden reduction curves obtained for a cohort of 22 patients as a function of the patient mean absorbed dose. Patients 3 (*) and 4 (**) are indicated.

The metastatic burden reduction curves for the 22 patients are shown in figure 2. The PMAD_MBR=0.5_ was 89 Gy (IQR: 83–105 Gy) across all patients. For cut-offs of 0.9 and 0.99 in the TCP calculation, PMAD median values of 88 Gy (IQR: 81–106 Gy) and 93 Gy (IQR: 89–111 Gy) were obtained respectively. An *α* parameter of 0.25 Gy^−1^ reduced the PMAD_MCP=0.95_ by 31% on average, with a median of 61 Gy (IQR: 57–73 Gy).Steep gradients were observed for 20 of the 22 patients, which show the potential for a large increase in metastatic burden reduction for a small increase in the patient mean absorbed dose delivered. Examination of the individual disease distribution for patients 3 and 4, showed that both had the highest variability in lesion absorbed doses within the patient cohort.

### Clinical dosimetry

Our previous study found a total of 379 lesions identified in 22 patients with a median of 11 lesions (IQR: 3–25) and 227 ml of metastatic tumour burden (IQR: 81–300 ml) per patient. The median PMAD_delivered_ across all patients was 19 Gy (IQR: 14–23 Gy) or 3.72 Gy GBq^−1^ (IQR: 2.80–4.69 Gy GBq^−1^). Further details can be found in Denis-Bacelar *et al* (2017).

### Comparison between predicted and delivered absorbed doses

**Figure 3.**
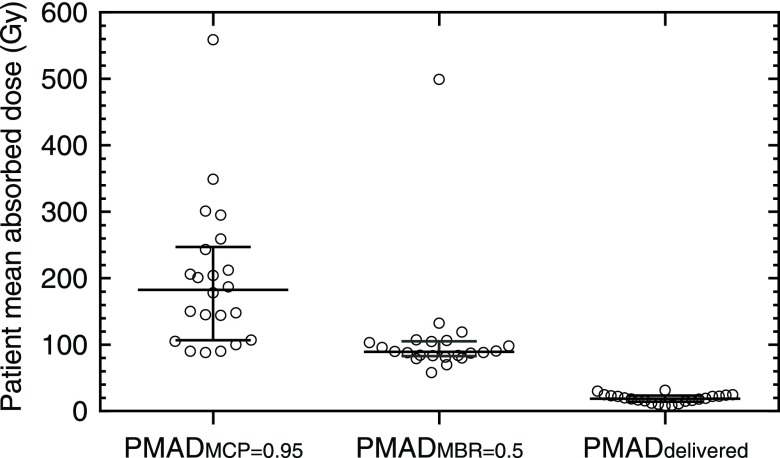
Range of predicted PMAD_MCP=0.95_ and PMAD_MBR=0.5_, and delivered PMAD_delivered_ for the 22 patients. The median and inter-quartile range is shown.

Table 1 and figure 3 summarize the results obtained for the patient cohort studied. The median PMAD_MCP=0.95_ was 183 Gy (IQR: 107–247 Gy), the PMAD_MBR=0.5_ was 89 Gy (IQR: 83–105 Gy) and the median PMAD_delivered_ dose was 19 Gy (IQR: 14–23 Gy).

**Table 1.** The patient mean absorbed doses delivered (PMAD_delivered_) and predicted to eradicate all the lesions (PMAD_MCP=0.95_) and to reduce the metastatic tumour burden by 50% (PMAD_MBR=0.5_) for the 22 patients. Absorbed doses for MCP values of 0.9 and 0.99 and TCP values of 0.9 and 0.99 for the MBR  =  0.5 calculation are shown between brackets. Absorbed doses for MCP  =  0.95 and MBR  =  0.5 with a radiosensitivity parameter *α* of 0.25 are shown in the last two columns. Patient no.

Patient no.	Patient mean absorbed dose (PMAD) (Gy)				
	Delivered	MCP = 0.95	MBR = 0.5	MCP = 0.95 (*α* = 0.25 Gy^−1^)	MBR = 0.5 (*α* = 0.25 Gy^−1^)
1	8	88 (85–93)	80 (79–90)	60	56
2	22	243 (237–256)	84 (81–87)	166	58
3	15	349 (340–367)	57 (58–64)	239	42
4	30	559 (545–588)	499 (486–500)	383	331
5	22	206 (200–218)	79 (79–85)	140	56
6	11	105 (102–111)	70 (65–72)	72	48
7	31	295 (287–312)	84 (81–89)	201	57
8	20	212 (207–224)	105 (101–110)	145	71
9	25	187 (182–198)	88 (86–93)	128	60
10	18	100 (97–105)	87 (84–92)	68	60
11	19	201 (196–212)	106 (106–111)	138	74
12	15	107 (104–113)	90 (88–91)	73	59
13	24	144 (140–150)	132 (129–141)	99	92
14	9	178 (174–187)	119 (114–122)	122	79
15	23	145 (142–153)	103 (102–109)	99	72
16	10	90 (88–95)	88 (88–93)	62	62
17	18	150 (146–157)	107 (105–112)	103	74
18	25	204 (199–216)	90 (88–95)	140	62
19	22	301 (293–319)	81 (80–86)	205	55
20	17	259 (252–273)	98 (95–104)	177	66
21	16	148 (144–155)	96 (94–99)	101	66
22	12	90 (88–95)	83 (84–90)	62	60

## Discussion

Standard radiobiological models of metastatic control were used to assess the patient-specific absorbed doses required to cure all metastatic bone lesions in 22 patients with CRPC metastatic to bone. A new treatment planning framework was introduced to predict the decrease in metastatic burden as a function of the absorbed doses delivered, which could enable personalised treatment of bone metastases with the aim of prolonged survival.

The results showed a strong correlation between metastatic control probability and the degree of variability of the absorbed doses delivered to multiple lesions, with a median PMAD_MCP=0.95_ of 183 Gy. A strong correlation between metastatic control probability and minimum lesion absorbed dose was also observed. This indicates the limitations of this model alone for treatment planning, since a metastatic burden reduction, rather than complete eradication of metastases can still be considered a good response to treatment. Application of the newly proposed method to the clinical data showed that a median PMAD_MBR=0.95_ of 89 Gy would be required to reduce the metastatic tumour burden by 50% in this patient cohort. The patient-specific metastatic burden control curves showed that 91% of the patients have a steep gradient of the curve, where a large benefit in terms of metastatic burden reduction could be achieved by a small increase in the patient mean absorbed dose. In 50% of patients, the patient mean absorbed doses required to reduce the metastatic burden by half varied by only 21 Gy, whilst differences of 119 Gy were found necessary to eradicate all the lesions. The metastatic burden control model could be used to identify patients that would benefit more from treatment. For instance, the results for patient 4 suggest that this treatment may not be effective, as it would require very high patient mean absorbed doses to obtain any significant metastatic reduction, which would likely result in myelotoxicity. To reduce the risk of toxicity, treatment could also be planned according to the lesions that for a given administered activity receive high absorbed doses and the lesion(s) with low absorbed doses could be treated with a boost dose of external beam radiotherapy.

The predicted absorbed doses for elimination of metastases and metastatic burden reduction were considerably higher than the calculated delivered absorbed doses. However, a wide range of lesion absorbed doses in patients treated with ^186^Re-HEDP are available in the literature. Israel *et al* obtained a mean absorbed dose delivered to lesions of 2.1 Gy GBq^−1^ from 1.532 GBq (Israel *et al* 2000) in close agreement with our results. Higher absorbed doses were obtained by Maxon *et al*, with 33 Gy GBq^−1^ from 1.225 GBq calculated using whole-body planar imaging (Maxon *et al* 1990); and by Andreou *et al* with 52 Gy GBq^−1^ from 1.295 GBq (Andreou *et al* 2010). Differences in lesion absorbed doses are likely due not only to the challenges associated with the heterogeneity of uptake of bone-seeking radiopharmaceuticals and the difficulty to calculate the tumour volume (Bouchet *et al* 1999, Bouchet and Bolch 1999, Strigari *et al* 2007), but also to the lack of standardization of imaging and dosimetry protocols. Nonetheless, the calculations were based on the relative distribution of absorbed doses delivered to multiple lesions in a given patient. Therefore the methodology and conclusions drawn from this study are not affected by the absolute absorbed doses. Absolute dosimetry would be required to fully understand the biological effects of the radiation doses delivered and tailoring the treatment to individual patients (Pouget *et al* 2015). Therefore more efforts are required towards improving the reproducibility and repeatability of the absorbed dose as a biomarker of response in molecular radiotherapy.

This study has some limitations. The relative biological effectiveness (RBE) and radiosensitivity parameters vary for different radionuclides, tissues and activity distributions. The uptake of radiopharmaceuticals used for bone metastases highly depends on the osteoblastic activity for bone-seeking agents and is highly heterogeneous at the microscopic scale, particularly for alpha emitting radionuclides. However the limited resolution of clinical imaging systems only allows for macroscopic dosimetry and the assumptions of uniform distribution of activity are often made for bone lesion dosimetry (Liepe *et al* 2003, Baum *et al* 2016, Pacilio *et al* 2016). The parameters used in the LQ model have been derived from uniformly distributed absorbed doses delivered by external beam radiotherapy in prostate cancer patients, as these are not available for mCRPC and ^186^Re-HEDP. Different radiobiological parameters would have the effect of shifting the absorbed doses required to produce a given effect to lower or higher values although the relative positions of the curves would remain constant for all patients. A change in *α* from typical prostate cancer cells (0.15 Gy^−1^) to less radio-resistant cells (0.25 Gy^−1^) would reduce the patient mean absorbed dose required to kill all the metastases and half the metastatic tumour burden by 31–32%. Further clinical studies with pre- and post-therapy imaging would allow the validation and refinement of the parameters used in metastatic burden reduction model.

The use of bone-seeking radiopharmaceuticals is rapidly increasing. A survival benefit has been observed in patients treated with ^223^Ra as compared to placebo and ^177^Lu-DKFZ-617 PSMA has been shown to induce remission in a patient with metastatic prostate cancer (Kratochwil *et al* 2015). Due to the low number of patients treated and the wide range of absorbed doses reported from fixed and/or weight-based administered activities, absorbed dose response relationships have not yet been established. Radiation therapy can be an effective treatment for bone pain relief and 48 Gy in 3 fractions SBRT treatment in patients with multiple oligometastases, have shown promising long-term disease control (Salama *et al* 2012). An on-going clinical trial is investigating the eradication of oligometastases, including bone lesions, in patients with mCRPC (NCT02685397). Radiation therapy is typically used in symptomatic and late stages of disease. However, implementation of treatment early in the course of the disease may increase quality of life and reduce overall metastatic tumour burden; in turn, delaying disease progression and improving long-term survival. This could be of particular relevance for molecular radiotherapy, where radiation is targeted to specific molecular sites and toxicity is limited.

## Conclusion

The metastatic burden reduction model proposed in this study offers the potential to provide individualised treatment and could be used as a patient selection tool by relating potential benefit and toxicity. Metastatic burden reduction curves could be predicted from pre-therapy imaging or from therapy imaging, which would enable adaptive treatment planning for repeated administrations. This simple tool has the potential to inform patient management by individualising treatment in patients with multiple tumours or metastatic disease undergoing molecular radiotherapy. Future studies of treatment response will allow validation and refinement of the model.
